# Advances in Material Modification with Smart Functional Polymers for Combating Biofilms in Biomedical Applications

**DOI:** 10.3390/polym15143021

**Published:** 2023-07-12

**Authors:** Luis Alberto Mejía-Manzano, Patricia Vázquez-Villegas, Luis Vicente Prado-Cervantes, Kristeel Ximena Franco-Gómez, Susana Carbajal-Ocaña, Daniela Lizeth Sotelo-Cortés, Valeria Atehortúa-Benítez, Miguel Delgado-Rodríguez, Jorge Membrillo-Hernández

**Affiliations:** 1 School of Engineering and Sciences, Tecnologico de Monterrey, Monterrey 64700, Mexico; alberto.mejia.m@tec.mx (L.A.M.-M.); paty.vazquez@tec.mx (P.V.-V.);; 2 Institute for the Future of Education, Tecnologico de Monterrey, Monterrey 64700, Mexico; 3 EVONIK, México, Av. Calzada México-Xochimilco 5149, Ciudad de México 14610, Mexico

**Keywords:** biofilms, smart polymers, biomedical devices, health-associated-diseases, prevention

## Abstract

Biofilms as living microorganism communities are found anywhere, and for the healthcare sector, these constitute a threat and allied mechanism for health-associated or nosocomial infections. This review states the basis of biofilms and their formation. It focuses on their relevance for the biomedical sector, generalities, and the major advances in modified or new synthesized materials to prevent or control biofilm formation in biomedicine. Biofilm is conceptualized as an aggregate of cells highly communicated in an extracellular matrix, which the formation obeys to molecular and genetic basis. The biofilm offers protection to microorganisms from unfavorable environmental conditions. The most frequent genera of microorganisms forming biofilms and reported in infections are *Staphylococcus* spp., *Escherichia* spp., and *Candida* spp. in implants, heart valves, catheters, medical devices, and prostheses. During the last decade, biofilms have been most commonly related to health-associated infections and deaths in Europe, the United States, and Mexico. Smart, functional polymers are materials capable of responding to diverse stimuli. These represent a strategy to fight against biofilms through the modification or synthesis of new materials. Polypropylene and poly-N-isopropyl acrylamide were used enough in the literature analysis performed. Even smart polymers serve as delivery systems for other substances, such as antibiotics, for biofilm control.

## 1. Introduction

Infectious diseases, caused by various biological agents as part of a natural cycle, have always been a part of human life. However, in recent years, the World Health Organization (WHO) has reported an increase in the incidence of infectious diseases, especially in low and middle-income countries. This is largely due to inadequate sanitation, limited access to healthcare, low vaccination rates, vectors, poverty, and malnutrition [1]. This has also impacted high-income countries, as happened in the COVID-19 pandemic.

Nosocomial infections, or healthcare-associated infections, occur during or post-medical care, absent in patients when they are admitted to hospitals or healthcare facilities [2]. Some factors for nosocomial infections are environment, patient condition, susceptibility, and lack of awareness among staff and healthcare providers. It is not difficult to predict that the source of these causing agents can be patient microflora, other patients, staff, and the environment (equipment, materials, food, water, or hospital wastes) [2].

Many of these human pathogens are bacterial microorganisms forming biofilms as a survival mechanism. It has been indicated that the chronicity of these diseases is closely related to the biofilms [3]; hence combating biofilm formation or proliferation in the health area is a topical issue.

The general strategies for combating biofilms encompass diverse options according to the stage of biofilm infection and the context of colonization. Some of these strategies are directed to prevention, some to control, and others to treatment. The preventive measures consider hygiene, the use of antimicrobial agents, the use of dispersing agents or molecular-based strategies (blocking of the cell-to-cell communication or quorum sensing, interference with the key molecules biosynthesis, enzyme-mediated control, interference with receptor proteins by antagonists, degradation by electrochemical methods), use of non-pathogenic bacteria as competitors, surface modification or use of novel surface materials [4,5]. For treatment of already present infections, when these involve a foreign body (like devices), flow management cleaning or mechanical removal may help the issue. Still, when these are impossible, antibiotic administration of some other substances or alternative mechanisms must be considered [6,7].

This literature review presents the state-of-the-art in using smart polymer materials to prevent and control biofilm formation in the biomedicine area, attending to the branch of surface modifications or novel materials. The questions it pretends to answer are: What is a biofilm? Which microorganisms have been reported to form biofilms in the biomedical area? Which diseases have been associated with these? Which is the importance of biofilms in health? What is known about the current biofilm formation processes? What is a smart, functional polymer? Which have been the main applications of functional polymers? What has been done in using smart polymers to combat/control biofilm formation? Finally, general conclusions are summarized, and trends in applying these polymers for disease-based problems are described.

## 2. Relevance of Biofilms in the Biomedical Field

### 2.1. Biofilm Conceptualization

The microorganism’s organization can be classified into planktonic and biofilms. The first one is less frequent in nature (estimated as less than 0.1% of the bacterial population), in which microorganisms live in an isolated and free conformation in a medium [8]. But, around 80% of the microbial population are organized more complexly, known as biofilms [8]. Biofilms have been described as “An aggregate of microorganisms in which cells are frequently embedded within a self-produced matrix of extracellular polymeric substances (EPS), adhering to each other and to a surface” [9]. The biofilms are visualized as communities of microorganisms since they are a set of various species that interact in the same system, wrapped in a matrix in cell-cell and surface-cell contact. Generally, the lower layer of the biofilm presents contact with a substrate or is attached to a biotic or abiotic surface [10].

When biofilms started to be studied, these were defined as a ”dense cover of bacteria cells embedded in a confluent biomatrix" [11], which had a lower percentage of viable cells per area unit (cm^2^) and grew more slowly and remained in a stationary state, compared to planktonic cells that have a logarithmic phase of growth and possess a higher percentage of viable cells [11]. Later, this concept was enriched with information obtained through novel visualization techniques such as Confocal Scanning Laser Microscopy (CSLM), which gave data about the heterogeneity of the structures showing variable cellular distribution, presence of extracellular polymers and water channels but also empty spaces [12]. The tendency to form aggregates in different places depended on the bacterial species. Nuclear Magnetic Resonance Imaging (NMRI) and Particle Image Velocimetry (PIV) allowed visualizing the convective flow of substrates in water channels within the biofilm [12]. In the same way, using microsensors for studying biofilms presented unequivocal evidence of oxygen gradients that allows the existence of mixed species in the biofilm [12]. In 2010, it was found that less than 10% of the components of a biofilm are dry mass, while about 90% is made up of the extracellular matrix [13]. The composition of sessile cells within the matrix is heterogeneous, with irregular distribution (mixed species) covered with the matrix of EPS. This extracellular matrix was reported to be formed mainly of insoluble polysaccharides like alginate, proteins, lipids, flagella, pili, and extracellular DNA (eDNA) [14]. Biofilms represent a microbial organization resulting from an evolutionary defense mechanism by organisms to protect themselves from unfavorable environmental conditions outside the extracellular matrix. Therefore, the formation of microcolonies or macro-colonies encapsulated in the extracellular matrix provides properties and functions of resistance, tolerance, and persistence to biocides and antibiotics, as well as communication and interaction factors allowing exchange capacities of genetic and molecular material [10]. The success of pathogens microorganisms forming biofilms depends on their rapid adhesion to survive in hostile environments. EPS becomes a medium in which nutrients are distributed. In the same way, EPS works to acquire nutrients from the external environment, possibly with its sorption ability. This allows the sessile cells to grow in a less stressful environment [15].

### 2.2. Statistics about Biofilms in Disease

Microorganisms forming biofilms may infect and develop rapidly in materials for treating diverse illnesses. Many of these materials, like medical implants, may be present with patients for the rest of their lives. The bacteria forming these biofilms come from the patient’s skin, environment, or healthcare personnel [16]. In the case of implants, once the bacterium has adhered to the implant surface and formed the biofilm, this biofilm acts as a source of infection, especially in immunocompromised patients, compromising the patient’s life [16].

In this sense, evidence indicates how biofilm is related to chronic infections using medical devices. Cámara et al. recently performed research on the global biofilm infections and associated healthcare burden, showing the following facts [17]:

◦ Most chronic wounds have a biofilm (78.2%). In 2017, healthcare spent 7800 billion dollars on injuries, with 281 billion dollars linked to biofilms.
◦ Biofilms impact the lung and digestive systems of cystic fibrosis patients. They are resilient to antibiotics and contribute significantly to the economic impact of CF, with global costs reaching 7509 million dollars annually.
◦ Infective endocarditis is a serious condition caused by bacteria or fungi. Biofilms are a major cause of the disease. The incidence of infective endocarditis is increasing, with an estimated annual economic impact of $16 billion globally.
◦ Bacterial biofilm infections cause chronic sinusitis and can be treated with antibiotics or surfactants. The global cost of this condition is estimated to be $24.4 billion.
◦ Biofilms are a recurring issue in catheter-associated infections. The cost of diseases due to central venous catheters is estimated at 11.5 billion dollars globally, while catheter-associated urinary tract infections affect around 150 million people annually, costing $1 billion worldwide.
◦ Biofilm infections in ophthalmology can occur in the eye, eyelids and as a result of contact lenses or artificial lenses introduced during cataract surgery. Eye conditions associated with biofilms cost around $759.3 million annually globally.
◦ Although pacemaker and defibrillator insertion are generally considered low-risk, biofilm infections affect 1–3% of patients within 12 months, leading to antibiotic treatment and often device replacement. Global spending on biofilm-related illnesses in pacemakers and defibrillators is $220 million annually.
◦ Endotracheal tubes on ventilated patients can develop bacterial biofilms within 24 h of intubation, increasing the risk of ventilator-associated pneumonia. 35% of ICU beds in the USA are occupied by mechanically ventilated patients, costing an estimated $920 million globally annually.
◦ Prosthetic joint surfaces can get infected with biofilms, leading to antibiotic failure and possibly surgery. There are few alternatives to surgery, and the cost of revision surgery due to biofilm infections is $7849 million globally annually.

Biofilms in healthcare are a major cause of microbial infections globally, with over 65% to 80% being resistant to standard anti-microbial treatments, according to the National Institute of Health [18]. In Mexico, nosocomial infections in public health institutions occurred in patients with diseases, mainly pneumonia, and high urinary tract infections [19], followed by surgical wounds. A recent study suggested biofilms were responsible for 65% of nosocomial infections in this country [20]. As an example, in 2016, 204 patients with a CVC (Central Venous Catheter) between 4 and 6 years under critical condition were monitored after placing this device in the intensive care area for seven days, taking two blood cultures daily (one through this catheter and another through the peripheral vein) [21]. The cultures were analyzed using a MicroSan autoSCAN-4 System, revealing the presence of microorganisms in the blood cultures of 24 patients, of which nine were not related to the catheters and 15 were related to colonized catheters. *Staphylococcus* spp., *Candida albicans, Escherichia coli*, and *Stenotrophomonas* spp. were found in the cultures. The researchers concluded that the contamination had an external origin and was not caused by commensal germs on the skin [21].

In the European Union in 2009, it was estimated that about 50,000 deaths per year and 3 million patients had biofilm-derived infections, affecting various organs (22%) or producing pneumonia (15%) and septicemia (14%) as a consequence, the other 50% responded to common cancer and circulatory diseases followed by respiratory diseases and external causes of death [22]. Countries like the United States reported 99,000 deaths per year and 1.7 million processes related to biofilms in 2008 [22]. For the current year, 2023, approximately 5% (722,000 cases) of hospital admissions have been estimated as healthcare-associated infections by the U. S. Centers for Disease Control and Prevention (CDC), with a result of 75,000 deaths [23].

### 2.3. Common Bacteria Forming Biofilms and Medical Devices

The most common biofilm-forming microorganisms in medical devices are listed in Table 1. The table also lists the infections caused by each biofilm, mainly surgical site infections, urinary tract infections, pneumonia, and yeast infections.

*Staphylococcus aureus* is commonly found on the skin and mucous membranes of animals. The World Health Organization (WHO) has ranked the methicillin-resistant strain in the global priority list of antibiotic-resistant pathogens. It is one of the main causes of osteomyelitis, soft tissue, and skin infections, delaying healing processes. This biofilm affects the skin barrier function and elicits inflammatory responses involving interleukins (IL) IL-17, IL-12, and IL-6 [24]. For their part, *Escherichia coli* is a rod-shaped bacterium, a facultative anaerobe that lives as a commensal in the mammalian gut and causes disease at specific sites. Its biofilm gives *E. coli* resistance to desiccation and bleach; this happens thanks to the production of the matrix components like cellulose [25]. *Pseudomonas aeruginosa* has a high range of adaptability to the microenvironment, forming biofilms with high antibiotic tolerance. As an important number of medical equipment is used in patients with assisted ventilation, part of the *P. aeruginosa* biofilm can access the respiratory tract and create infections [26]. Another pathogen forming biofilms is *Staphylococcus epidermidis*, which produces slime enhancing its attachment to different surfaces and acting like “cement” for other bacteria [26]. *S. epidermidis* has cells within the biofilms embedded in the exopolysaccharide matrix. This bacterium can give nosocomial infection that causes coagulation problems in implanted devices (endocarditis, bloodstream, and central catheter infections) [27]. In *Enterococcus faecalis*, its enterococcal surface protein lets it adhere and colonize abiotic surfaces, while its gelatinase, an extracellular metalloprotease, hydrolyzes collagen and hemoglobin, favoring bacterial adherence [28]. The biofilm of *Klebsiella pneumoniae* is second only to *Escherichia coli* in nosocomial Gram-negative bacteremia, often more resistant to antibiotics and host defenses compared to its planktonic form, making it difficult to treat, and it can be found in various medical devices such as catheters, ventilators, and surgical instruments [30]. Finally, *Candida albicans* biofilms are resilient and great adherent bacteria communities, benefiting fungal adhesion and protecting against external threats. These are particularly concerning because they can cause infections in individuals with compromised immune systems, such as those undergoing medical procedures or chronic illnesses. Like previous bacteria, it is resistant and found in catheters, dentures, and prosthetic devices [29].

## 3. Molecular and Biological Bases of Biofilm Formation

**Figure 1:**
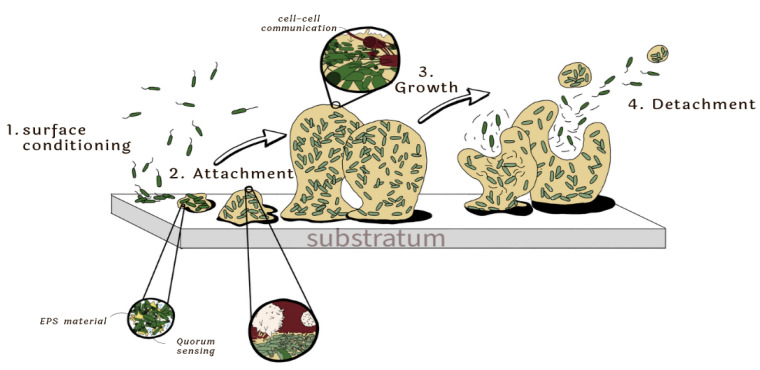
Typical phases in the formation of a biofilm.

The phenomenon of adhesion of bacteria to surfaces has been studied for a long time. Traditionally, three phases are known in the biofilm formation process: adhesion, proliferation or maturation, and detachment [31,32] (Figure 1). The description of distinct mechanisms of biofilm formation (attachment stage) for different microorganisms is found in Table 2. Biofilm maturation and dispersal mechanism can be consulted in the correspondent references. Each phase is important in the survival race of the bacteria within the system, and it is possible to find factors that determine the biofilm extension depending on the microorganisms present, the shear forces experienced, the temperature, and the nutrient availability in the environment [13].

The first step is a reversible process and is crucial for biofilm formation. Bacterial cells can attach to any surface that provides a substrate. This requires the initial adsorption of macromolecules to an inert surface [36]. Subsequently, there must be proximity between the cells and the surface. This is achieved by contact with a contaminating material or fluid on the surface [37]. Adhesion can be non-selective (abiotic surfaces) or specific (biotic surfaces such as cells, skin, or plant material) [34,36,38]. Once in contact, the cells, by chemotaxis (signaling that generates movement due to the gradient of molecules), move toward the nutrient source. In a matter of minutes, a series of physical phenomena occur that generate intermolecular non-covalent weak attractive and repulsive forces such as hydration, electrostatic, hydrophobicity, or physicochemical interactions such as van der Waal, which compete with bacterial movement and shearing effects of some fluid on the surface [32,37,38].

It has been found that, at this stage, characteristics of the surface (inert or abiotic), such as roughness, hydrophobicity, surface charge, and the presence of films of other chemical compounds, condition the speed of adhesion; generally rough, positively charged, and hydrophobic surfaces facilitate colony attachment [37]. However, this depends on the bacterium’s type and state and the medium’s zeta potential [36]. The distance between bacteria and surfaces is also critical. Distances between 10–20 nm generate a repulsion [32,36].

Cell adhesion becomes irreversible due to dipole-dipole interactions and hydrogen, ionic or covalent bonds, which occur in a shorter range. This is facilitated by adhesin molecules (cell wall glycoproteins, such as AB proteins that bind to *Staphylococcus aureus* fibrinogen, Alpha lectin from *saccharomyces cerevisiae*, or the Als family of proteins from *C. albicans*) [33], together with specific proteins (fibrinogen-binding protein Fbe/Sdrg, fibronectin-binding protein Embp), enzymes (autolysins, GehD lipase), proteinaceous surface organelles such as flagella and pili/fimbriae [32]. These molecules are generally antigens with a polysaccharide nature that bind and interact with molecules on cell membrane surfaces or surfaces modified by nutrient deposition. Subsequently, the bacteria begin to produce chemicals (acryl homoserine lactones in case of Gram-negative and self-induction peptide or oligopeptides such as furanosyl borate diester in case of Gram-positive, known as autoinducers, since it controls the expression of genes of the same type of cells or between different species) [33] that generate a chain reaction to produce the same polysaccharides. This phenomenon is known as Quorum sensing, a process by which bacteria communicate chemical signals to mediate physiological processes [38,39].

Extracellular polysaccharides (EP) (consisting of polysaccharides, proteins, DNAs, lipids, and polymeric compounds) play an essential role in water and nutrient retention, genetic exchange, and intercellular communication. Depending on the molecules that make it up, EPs can be neutral or anionic (uronic acid and ketal-lined pyruvates in Gram-negative) [37] or cationic (partially acetylated 1→4 glycosidic linkages of N-acetyl galactosamine and N-acetylglucosamine, or Pel, in Gram-positive) [40], and this can condition the attachment to different structures, considering the type of material. Inside the cell, the Cyclic dimeric guanosine monophosphate (c-di-GMP) is a nucleotide base that has been the most studied signaling molecule responsible for mediating biofilm formation via gene expression [41]. Diguanylate cyclase enzymes catalyze the formation of c-di-GMP, and specific phosphodiesterase enzymes catalyze its degradation [35]. In some cases, concentrations greater than 200um of s-di-CMP reduce biofilm formation [36]. Also, sRNAs, noncoding RNAs, have been studied as QS molecules [38]. At this stage, the cells form monolayers through replication and attraction of other cells [33]. The QS molecules concentration modulates bacterial population density by controlling gene expression [35].

Once the autoinducers reach a certain threshold, the genetic machinery of the cells indicates the multiplication of the cells to form a microcolony, which continues to grow and generate changes in the expression of the genes that modify the structure of the EP to go from a state of agglutination to a state of dispersion or detachment when the pattern of gene expression differs significantly between sessile cells and free cells that adhere to the biofilm. Although the cells are detached from the rest at this stage by changes in the environment, autolysis, nutrient starvation (hindered nutrient transfer), or external forces, biofilms remain maintained by the action of autoinducers or other processes [37]. Biofilm erosion or sloughing is vital for recovering aging biofilm activity [36].

## 4. Smart, Functional Polymers

Smart, functional polymers are also called stimuli-responsive polymers because of their capability to respond to external stimuli. This reversible or irreversible response may be achieved through color, light, conductivity, or another property change [42] derived from structure, stability, and function alterations [43]. The stimuli of the polymers may be as diverse as pH, ionic strength, cosolvent composition, humidity, electric/magnetic/sound fields, light intensity, temperature, and mechanical stress [42,43,44].

### 4.1. Classification of Smart Polymers

The classification of smart polymers is diverse and depends on the criteria. The most basic division attends to the nature of the stimulus, identifying polymers responding to physical, chemical, and biological stimuli [42,45]. Also, a classification has been proposed based on the number of stimuli these materials respond to with single-stimulus and multi-stimulus responsible polymers. As examples of multi-stimulus polymers can be mentioned thermos-photochromic acrylamide-azobenzene materials, poly(2-dimethylamino) ethyl methacrylate with pH and thermo-behaviors, and synthesized polymers containing thermo, light, and pH or redox responsible moieties [42]. Conversely, smart polymers may also be classified according to their physical form in linear free chains, covalently cross/linked gels, and reversible or physical gels and chain adsorbed or surface/grafted forms [46]. In the linear chains, it is said that the polymer undergoes a reversible collapse after applying an external stimulus. At the same time, covalently cross-linked gels are microscopic or macroscopic networks with swelling properties. Chain-adsorbed or surface-grafted forms are membranes or surfaces where the polymer swells or collapses on a surface, converting the interface from hydrophilic to hydrophobic and vice versa [46].

A more specific class of smart polymers is shape memory polymers, which can “memorize” a temporary or permanent shape and recover it after being deformed by an external stimulus [44,47]. The methacrylic acid ester, polyethylene, and some urethane-based polymers are typical examples. Based on molecular structure memory, polymers are subclassified into Types I, II, and III. Type I are physically cross-linked networks, Type II are chemically cross-linked, and Type III are multi-network systems, linked physically or chemically in a hybrid way, integrating composites or other structures or materials such as nanoparticles, dyes, or conductive materials [44]. For their part, Liu and collaborators established four types of shape memory polymers considering glass or melting transitions due to these materials may be amorphous or crystalline correspondingly. So, these four classes are I, II, III, and IV or covalently cross-linked glassy thermoset networks, covalently cross-linked semi-crystalline networks, physically cross-linked glassy copolymers, and physically cross-linked semi-crystalline block copolymers, having the first two the glass or melting transitions as switches and the third and fourth both glass or melting transitions as switch [48].

### 4.2. Main Applications of Smart Polymers

**Figure 2:**
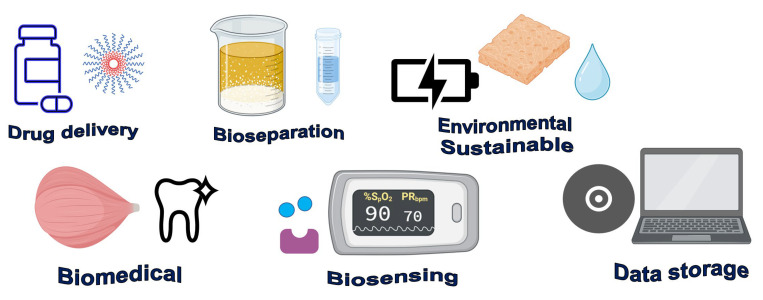
Main applications reported for smart polymers.

The diverse applications of smart, functional polymers are displayed in Figure 2. Drug delivery is one of the most benefited areas because drugs must be delivered in a controlled way in a precise location and at a specific time [49]. When smart polymers are included, release control may be modulated according to certain disease symptoms (fever or acidosis, for example) by sensing these changes [45]. Among the smart polymers used for drug delivery, it can be mentioned ethyl cellulose/poly(vinyl-alcohol)-poly(ethylene glycol) graft copolymer, N-isopropyl acrylamide and acrylic acid, and hydrogels of poly(nitrophenyl methacrylate-co-methacrylic acid) for gastrointestinal drugs [50] but a more extensive list of these polymers and their use is found in the review of James and collaborators [51]. In biomedicine, there is a vast number of applications for new materials. Particularly in tissue engineering, the substrate surface is key for the right cellular stages that this involves (adhesion, migration, and proliferation). Surgery medical devices are considering shape-memory polymers minimizing invasion [49], and even artificial muscles based on polypropylene (PP), ultra-high molecular weight polyethylene (UHMWP), and polyacrylonitrile (PAN) fibers have been synthesized [47]. Dentistry demands materials with sealing and obturating properties, which are an opportunity for smart materials [52]. More specific applications of smart polymers in disease prevention or management will be addressed in point 5.

Polyethylene glycol (PEG) derivates and temperature-sensitive Poly-N-isopropyl acrylamide (PNIPAAm) have been employed as reactive for the bioseparations or purification processes such as membrane operations, affinity precipitation, aqueous two-phase systems, and thermosensitive chromatography [46,49,53]. The detection and quantification of biological analytes or biosensing also exploit the properties or stimuli response polymers, for example, poly-(N-isopropyl acrylamide)-co-methacrylic acid (pNIPAm-co-MAAc) combined with surface plasmon resonance (SPR) let to recognize antibodies, pNIPAm with Au nanoparticles indicate elevate temperatures, poly(N-(3-amidino)-aniline) (PAAN) covering Au nanoparticles sensed CO_2_ concentrations and pNIPAm-co-acrylic acid served as low-temperature indicator of water [54]. In the environment and sustainable solutions fields, it has been reported that the binder in rechargeable batteries is made of polymers to make them efficient [49]. In this same via, water obtention from humid air is possible through a sponge-like made of poly-(N-isopropyl acrylamide) (PNIPAAM), mimicking natural water-capturing materials such as spider silk or carapace of Stenocara or desert beetle. Another example of the sustainable application of PNIPAAM and PNIPAAM-polystyrene is the elaboration of anti-fouling coatings on glass [42]. In information and communication technologies, high-capacity data storage devices are produced with 2D discs with azobenzene chromophores [49].

## 5. Current Advances in the Use of Smart Polymers for Combating Biofilms

Table 3 shows other research works regarding smart, functional polymers for the control/prevention of biofilm formation in biomedical materials.

### 5.1. Antimicrobial and Antibiofilm Activities of Smart Polymers

Antimicrobial peptides (AMPs) have potential as an alternative to antibiotics for treating bacterial infections. They are effective at killing bacteria, and resistance to them is slow to evolve. Polymers with cationic and hydrophobic substituents have been found to resemble AMPs in their killing mechanism by binding to bacterial membranes and causing deformations that lead to bacterial death [67]. Antibacterial polymers have been developed for about two decades. The properties that affect their bactericidal and toxicity properties include charge type, molecular weight, and morphology. Antimicrobial polymers are attractive alternatives to antibiotics because they have a lower tendency to evolve resistance in bacteria [67]. Variations in backbone chemistry, biodegradability, and morphology have been explored to achieve good antibacterial activities with low toxicity. The main classes of selective antibacterial polymers include poly(alpha-peptides), poly(beta-peptides), polycarbonates, polynorbornenes, and polymethacrylates. Antimicrobial polymers are being studied for their efficacy and safety. However, limited selectivity and challenges with biofilm and persistent states of bacteria hamper translation to therapies. Combination therapies and progress in animal studies and realistic settings show promise [67].

In the study by Jansen et al. [55], a central venous catheter made of polyurethane covered with a poly-N-vinylpyrrolidone (PNVP) coating (“Hydrocath”) and loaded with the antibiotic teicoplanin prevented infection by *S. epidermidis* and *S. aureus* [55]. Catheters were immersed in a teicoplanin solution during a specific time (3, 24, 48, and 72 h) and temperature (20, 40, and 60 °C), with different antibiotic concentrations (3–20 mg/mL) and solvents (water, ethanol-water, and ethanol). Due to the hydrophilic surface coating of the “Hydrocath” catheter, teicoplanin was well absorbed by the surface layer. The results showed that this catheter could impede *S. epidermidis* and *S. aureus* biofilm formation for at least 48 h with teicoplanin at 20 mg/mL loaded in ethanol at 40 °C [55]. Ruiz and collaborators describe another polymer mediating molecule delivery. They tested new smart surfaces-modified PP, grafted with poly-N-isopropyl acrylamide (PNIPAAm) or poly-acrylic acid (PAAc), which were bonded to vancomycin [56]. The modified polymers in the test for methicillin-resistant *S. aureus* show a pH-dependent release rate (pH 7.4, 37 °C ) of vancomycin and anti-biofilm formation. Although proofs were developed with the isolated films, it was not discarded that the same take part in manufacturing prostheses or catheters.

For their part, Anderson and his team investigated the performance of hydrogels composed of cross-linked poly-2-hydroxyethyl methacrylate (pHEMA), modified in their surface with octadecyl isocyanate and loaded with norfloxacin. The results of the simulated medical implant showed that not only zero-order releasing profile of drugs be achieved from these hydrogels, but also the antimicrobial activity of norfloxacin was improved to eradicate both planktonic and biofilm organizations of *S. epidermidis* from the medical implant surface [57].

Researchers from Sapienza University developed new segmented polyurethanes (PU) characterized by a hard/soft domain structure, having the same hard domain but a variable soft domain. The *soft* domain was constituted by one of the following macrodiols at 50%: polypropylene oxide (PPO), polycaprolactide (PLC), and poly-L-lactide (PLA). The bacterial adhesiveness of *S. epidermidis* after 24 h was reduced for soft phases PLA and PCL, particularly for PLA; this was improved by the increase in the soft phase content [58].

The research group of Steffensen introduced a novel antibacterial approach involving an advanced composite material applicable to medical devices. These polymeric composites consisted of a hydrogel network of cross-linked poly (2-hydroxyethyl methacrylate) (PHEMA) embedded in a poly (dimethyl siloxane) (PDMS) silicone elastomer produced using supercritical carbon dioxide. As a result, devices containing 25% (*w*/*w*) hydrogel and loaded with ciprofloxacin displayed a strong antibacterial effect against *Staphylococcus aureus* bacterial colonization, and subsequent biofilm formation on the device material was inhibited for 29 days. The researchers conclude that the hydrogel/silicone composite represents a promising candidate material for medical devices that prevent bacterial colonization during long-term use [59].

### 5.2. Combination of Smart Polymers and Antimicrobial Agents

Doroshenko et al. found that highly-branched poly(N-isopropyl acrylamide) with vancomycin end groups (HN-PNIPAM-van) offers a new approach to limiting the development of corneal infections and has an adjunct antimicrobial material to treat bacterial attachment for biofilm formation in *Staphylococcus aureus*. In their work, HB-PNIPAM-van altered the biofilm structure on a surface, inhibited bacterial cell attachment, and reduced the bacterial load in a simulated cornea infection [61].

*Staphylococcus epidermidis* is well known to be one of the major causes of infections related to medical devices. Ricciardelli and collaborators modified one of the most widely used silicone-based-polymers, polydimethylsiloxane (PDMS), by adsorption of pentadecanal and its most promising derivative, pentadecanoic acid, on the PDMS surface. The most interesting result was the clear and strong biofilm inhibiting effect that both the coating showed against *S. epidermidis* RP62A, which was not related to the anti-adhesive properties of the coatings since the extent of the reduction in cell adhesion was not so obvious as to cause such a strong inhibition of biofilm formation. This might be attributed to the release of the molecules from the coating, probably acting as quorum-sensing modulators [62].

Slettengren and his group investigated the effect on the biofilm formation of a silicone oil-coated polypropylene plastic used in a new automatic urinometer. Regardless of the oil viscosity, silicone oil-coated polypropylene plastic surfaces significantly inhibited the biofilm formation of all tested Gram-negative and Gram-positive bacteria [63]. Silicone oil did not affect bacterial or *Candida albicans* growth. Curli fimbriae appear to be the main biofilm-promoting factor. Adding silicone oil leads to a 50% reduction in biofilm formation in curly-positive strains. Polypropylene plastic without oil had a better effect in preventing biofilm formation than polystyrene. Silicone oil-coated polypropylene plastic surface significantly decreases early biofilm formation by pathogenic bacteria, including extended-spectrum beta-lactamase (ESBL) producing and multi-drug resistance (MDR) *Klebsiella pneumoniae*, as well as *C. albicans*.

### 5.3. Smart Polymer Nanomaterials as Antimicrobial and Antibiofilm Agents

Nanomaterials represent an additional resource to be combined with smart polymers to improve the future performance of biomedical devices and treat biofilms. The physicochemical, electric, magnetic, optical, and quantum properties of the nanoparticles have contributed to their use as antibiofilm agents and their antibacterial activity. In fact, silver nanoparticles are recognized for their antibacterial activity against *P. aeruginosa* [68,69]. Gold and zinc oxide nanoparticles combined with phloroglucinol inhibited the early-stage biofilm of *P. aeruginosa* [70]. Other materials exhibiting antibiofilm properties in nanoparticles are selenium [71], magnesium oxide [72], and calcium-silicate [73]. Graphene is another material displaying antibiofilm materials, although the specific mechanisms of this activity are unknown [74]. Also, nanoparticles geometry tends to be a factor affecting the antibiofilm activity, as reported for the hexagonal boron nanoparticles with the growth inhibition of *S. mutants* 3.3 and ATC25125, *S. pasteuri* M3, *Candida* sp. M25 [75].

Immediate application of novel synthesized or modified polymer-based materials for combating medical biofilms is summarized in dental applications. The team of Holban has summarized several works dealing with polymer modifications for avoiding biofilm formation (particularly of *C. albicans*) in dental materials through methods such as composition modification, surface treatments, and coatings [76] with nanodiamonds, diverse polymers, or nanoparticles.

Polívková and collaborators did a review [60] in which they gave an overview of nanostructured antimicrobial agents, especially silver ones, used together with biocompatible polymers as effective antimicrobial composites in healthcare. Silver-coated catheters showed significant in vitro antimicrobial activity and prevention of biofilm formation. The multifaceted mechanisms of actions of Ag^+^ ions prevent the development of bacterial resistance, and, for this reason, nanostructures silver is an excellent candidate for the preparation of antimicrobial coatings for polymeric medical devices.

Recently, a coating system using copolymers composed of diethylene glycol methyl ether methacrylate and 2-hydroxyethyl methacrylate was developed by alternatively fabricating monomers layer by layer on a titanium surface. The antibiotic elution characteristics were investigated against *S. aureus* species using one to four layers of MLTRPB. As a result, both in vivo and in vitro assessments were demonstrated. Preventive effects in vitro studies were identified when more than four coating layers were applied, ensuring promising outcomes of the MLTRPB coating. Also, in this study, the temperature-responsive polymer brushes with a lower critical solution temperature (LCST) in the 38–40 °C range were polymerized and attached to the titanium implant surface. Multi-layered temperature-responsive polymer brush (MLTRPB) substantially decreased the bacterial bioburden on the implant, correlating with the number of layers. MLTRPB substantially decreased the bacterial bioburden on the implant, correlating with the number of layers again. Using a four-layer MLTRPB resulted in a three-log reduction in bacterial biofilms, which they considered significant in clinical practice [66].

### 5.4. Stimuli-Responsive-Smart Polymers as Anti-Biofilm Agents

The following examples are Among the smart polymers that have not been properly used in the treatment or biofilm control in biomedical devices yet, but which have shown interesting results in preliminary tests for being incorporated in the field in the near future. Wei and colleagues have described a set of surfaces constituted by a releasing unit and a killing unit. The first is mainly PNIPAAm, and the second contains diverse elements (quaternary ammonium salts, nanoparticles, or polymers such as poly(p-phenylene ethylene)). So, when the releasing unit detects a stimulus (pH, salt, or temperature change), a response in three ways can be generated: a load of a biocide, the killing of the bacteria, and the release/detachment of the bacteria, it was known as the “kill-release” strategy [77]. A polymeric coating with azobenzene demonstrated efficacy in biofilm detaching against *P. aeruginosa*, *E. coli*, *S. aureus*, and *S. mutants* sucrose independently. Azobenzene suffered a trans to cis isomerization (photo fluidization) due to their exposition to light [78].

Scientists have developed micelles that respond to visible light and deliver nitric oxide (NO) and antibiotics together for more effective antibiofilm treatment. They used a photo-responsive monomer to create NO-releasing amphiphiles, which self-assemble into micellar nanoparticles that release NO under visible light. The nanoparticles can also carry hydrophobic antibiotics like ciprofloxacin and release NO and antibiotics when irradiated with light. This approach shows promise for future biomedical applications [79].

In their work, Li et al., designed a pH-sensitive anti-biofilm nano system formed by self-assembly between negatively charged carboxyl groups of poly(ethylene glycol_-COOH-polyethylenimine-2,3-dimethylmaleic anhydride (PPD) and positively charged amines on the surface of carbon dots derived from the ashes of calcined l-lysine powder (CDLys) (PPD@CDLys for short). The copolymer PPD@CDLys can easily penetrate dense biofilms and become positively charged through hydrolysis. This leads to disassembling the nanosystem and creating a biocidal cationic polymer that kills bacteria in the biofilm. The released CDLys induces reactive oxygen species that degrade the matrix of extracellular polymer substances and kill bacteria deep within the biofilm. The nanosystem effectively inhibits biofilm formation and destroys mature biofilms through the synergy of cation and ROS. It also exhibits outstanding biocompatibility and can be an effective anti-biofilm agent for controlling bacterial infections [80].

Recently, Arshad et al., fabricated gentamicin sulfate-laden stimulus-responsive polymeric microarray patches to treat bacterial biofilms. The patches were made using vacuum micro molding and contained sodium hyaluronate, gelatin, polyvinyl alcohol, and D-sorbitol. Analysis showed the patches had uniform micro projections, were amorphous solids, and had non-covalent interactions between substrates and enzymes. The patches released 96% of the antibiotic within 150 min and had antibacterial effects against *S. aureus*, *P. aeruginosa*, *E. coli*, and *S. enterica*. The patches reduced S. aureus and P. aeruginosa biofilm biomass by at least 70% and healed S. aureus-infected wounds within 11 days. The patches can be used for on-site stimulus-responsive administration of gentamicin sulfate and managing *S. aureus*-infected cutaneous wounds [81].

## 6. Conclusions and Perspectives

Biofilms are bacterial communities in a biological matrix that interact to survive. In biomedicine, nosocomial infections and mortality have been correlated with biofilm formation at national and international levels. In Europe, USA, and México, up to 10% of disease events may be associated with biofilms. Biofilms have been found in medical implants, catheters, heart devices, orthopedic materials, and sectorized diseases. The stages of the biofilm cycle are attachment, growth, and detachment. Recent studies have indicated that bacteria secreted several biomolecules favoring the biofilm structure formation among exopolysaccharides, diverse proteins, lectins, lipids, and sugars. Each microorganism or community has mechanisms that regulate the adhesion of cells. Smart polymers are novel materials that can respond to determinate stimuli such as temperature, pH, light, or any other property, exploited in diverse fields. The panoramic of smart polymers indicates that these have been used to prevent and control biofilm formation. Synthetized polypropylene (PP)-based polymers are the principal support used, followed by Poly-N-isopropyl acrylamide (PNIPPAAm). Synergistic coupling of polymer modifications and the use of antibiotics have demonstrated improved inhibition of the biofilm formation in tests concerning the antibiotic, acting as regulated delivery systems. Another cause of the efficient antibiofilm activity of novel materials is their antifouling properties. Most of the reviewed works hold tests for a single biofilm-forming microorganism instead of several, so it is suggested to consider a wider spectrum of microorganisms to expand the possibilities in smart polymer applications.

## Figures and Tables

**Table 1:** Common biofilms reported in the biomedical industry, their descriptions, and the infections they cause.

Microorganism	Medical Device	Infections and Diseases	Reference
*Staphylococcus aureus*	Catheters	Osteomyelitis, soft tissue and skin infections, excessive inflammation, delayed wound healing, and re-epithelialization.	[24]
*Escherichia coli*	Urinary catheters	Gastroenteritis, sepsis, neonatal meningitis, and urinary tract infections.	[25]
*Pseudomonas aeruginosa*	Prothesis or orthopedic devices, heart valve infections, central catheters, endotracheal tubes	Burn infections, wound infections, pneumonia, and bloodstream infections.	[26]
*Staphylococcus epidermidis*	Artificial heart valves and prosthetic or orthopedic device	Endocarditis, bloodstream, and central catheter infections.	[27]
*Enterococcus faecalis*	Urinary catheters	Infections in the urinary tract, bacteremia, and endocarditis	[28]
*Candida albicans*	Catheters	Yeast infections, thrush, systemic candidiasis	[29]
*Klebsiella pneumoniae*	Ventilators	Pneumonia, urinary tract infections, bacteremia, and bloodstream infections.	[30]

**Table 2:** Distinct mechanisms of biofilm formation (attachment stage) for different microorganisms.

Microorganism	Mechanism	Reference
*Candida albicans*	Aggregation in the form of yeast. Al' glycoprotein family (Als1-7, 9) regulates adhesion to host cells or extracellular matrix proteins. Als3, Bgl2, Tec1p, Bcr1 zinc finger protein, RTA3, Hwp1, MAPK protein kinase, Sphingolipids with sterols and glycerophospholipids, as well as B-glucanase mannoprotein and target/encoding genes, promotes biofilm formation playing a pivotal role in the hyphal building. Ywpi deletion mutants adhere to polystyrene. Sfpt1 gene deletion enhances cell adhesion. Cells propagate, and germ tubes form to yield hyphae; mycelium growth causes colony fusing to form a monolayer with an extracellular matrix composed of polysaccharides and extracellular DNA (eDNA) that protects the cell inside the biofilm. eDNA induces cell lysis and QS.	[33]
*Streptomyces coelicolor*	A suitable source of nutrients y sensed by spores, they germinate under aerial growth, forming vegetative hydrophilic mycelia, which transitions to aerial hydrophobic hyphae with the extracellular proteinaceous surface formed by chaplins (ChpA-H) and rodlins (RdlA and RdlB). Fimbria structures play the same role in liquid-static cultures. CslA, SapB, and coding genes are also involved in attachment. GBLs, MMFs, Factor A, Factor-I, IM-s, VB, and PI factor act as QA molecules.	[34]
*Staphylococci (S. aureus*, *S. epidermidis)*	They attach to biotic surfaces containing fibrinogen, fibronectin, vitronectin, and collagen through cell wall-anchored proteins (FnBPA, FnBPB, ClfA-B, SdrC-H, and Brp) or to abiotic surfaces through hydrophobic and electrostatic interactions through extracellular glycopolymers (teichoic acids), autolytic enzymes (AtlA and AtlE) which are vital in the release of eDNA, and accumulation-associated protein (Aap or SasG) through its N-terminal A domain.	[31]
*Pseudomas aeruginosa*	Flagella, type IV pili, Cup fimbria, eDNA, and Psl polysaccharide initiate adhesion. Adhesin CdrA binds to Psl for aggregation. Cationic e-DNA cross-linking Pel and alginate exopolysaccharide enables biofilm formation, with lectins LecA/LecB and functional amyloid Fap. All are regulated by c-di-GMP, which also negatively regulates motility. LasI/LasR and Rh1I/Rh1R QS proteins act upon HSLs, and the 2-heptyl-3-hydroxy-4-quinolone system regulates rhamnolipid production for biofilm resistance to host immune response.	[35]
*Escherichia coli*	For initial attachment, Flagella and proteinaceous curly fibers (CsgA and CsgB) are required. Type 1 pili (FimA, FimH) and P pili (PapA, PapG). are central for irreversible attachment to mannose residues on cells. Cellulose, PGA eDNA, and colanic acid compose the biofilm matrix. Their production is regulated by c-di-GMP (primarily controlled by DgcE), negatively regulating motility. QS employs a furanosyl borate diester (AI-2) system.	[35]
*Acinetobacter baumannii*	These bacteria lack flagella. Csu pili and OmpA outer membrane protein mediate biotic and abiotic surface adhesion. Bap keeps the structure of the biofilm matrix. Synthesized alginate and PNAG exopolysaccharides together with eDNA ate biofilm matrix components. A Fe3+-induced AHL/AbaI/AbaR-based QS system increases the expression of Csu pili.	[35]

**Table 3:** Smart polymers used for treating or preventing bacterial biofilm formation in biomedical devices.

Polymer	Biomedical device	Microorganisms	Results	References
Poly-N-vinylpyrrolidone (PNVP) coating on polyurethane	Central venous catheter	*S. aureus*	Impeded biofilm formation for at least 48 h.	[55]
		*S. epidermidis*		
Polypropylene (PP) film crosslinked with PNIPAAm (PP-PNIPAAm) or PAAc (PP-PAAc)	Tentative components in prostheses and catheters	*S. aureus (MRSA)*	All vancomycin-loaded modified films showed significant anti-biofilm effects. The thinnest grafting layer of PP-PAAc had the greater reduction (91.7%)	[56]
Hydrogel of poly-2-hydroxyethyl methacrylate (pHEMA)	Intraocular lens surface	*S. epidermidis*	The antimicrobial activity of norfloxacin was improved to eradicate both planktonic and biofilm forms.	[57]
Polyurethane (PU) with soft domain constituted by one of the following macrodiols: polypropylene oxide (PPO), polycaprolactide (PCL), and poly-L-lactide (PLA).	Future synthesis/coating of medical devices	*S. epidermidis*	Best antifouling properties and reduced bacterial adhesiveness by PU-PLA (2 × 102 CFUs/cm^2^)	[58]
Hydrogel/silicone	Medical devices	*S. aureus*	Prevented the development of bacterial resistance during long-term use.	[59]
Nanostructured-silver-treated polymers	Catheters	*S. albus*	It prevented the development of bacterial resistance at 99.99% of *Pseudomonas aeruginosa* and *Staphylococcus albus*.	[60]
	Endotracheal tubes	*P. aeruginosa*		
	Wound dressings	*E. coli*		
	Surgical mesh	*B. anthracis*		
	Other polymeric fibers			
Poly (N-isopropyl acrylamide) with vancomycin end groups (HB-PNIPAM-van)	Infected dermal tissue model	*S. aureus*	HB-PNIPAM-van altered the biofilm structure on a surface, inhibiting cell attachment and reducing bacterial load.	[61]
Polydimethylsiloxane (PDMS)	Implanted medical devices	*S. epidermidis*	Strong biofilm inhibiting activity of the coatings, decreasing biofilm thickness for untreated and modified coatings.	[62]
Silicone oil-coated polypropylene (PP) plastic and polystyrene (PS)	Urinometer	*E. coli*, *P. mirabilis*	Silicone oil-coated polypropylene plastic surfaces significantly inhibited the formation of Gram-negative and Gram-positive bacteria biofilm. Even without PP, the plastic itself prevented biofilm formation more than PS.	[63]
Poly-4-hydroxybutyrate (PH4B)	Kinetted polypropylene implants	*E. coli*	Reduced biofilm formation on P4HB compared with PP flat films.	[64]
		*S. aureus*		
Smart hydrogel consisted of norspermidine, aminoglycosides, and oxidized polysaccharide	Medical devices	*P. aeruginosa*	Inhibition of the associated biofilm infections and chronic wound infections in clinics.	[65]
Multi-layered temperature-responsive polymer brush (MLTRPB)	Orthopedic implants	*S. aureus*	Antibacterial effect of the MLTRPB coating with more than four layers in vitro and in vivo studies.	[66]

## Data Availability

Not applicable.
